# MEX3A promotes colorectal cancer migration, invasion and EMT via regulating the Wnt/β-catenin signaling pathway

**DOI:** 10.1007/s00432-024-05845-9

**Published:** 2024-06-25

**Authors:** Jiannan Xu, Songyao Chen, Tengfei Hao, Guangyao Liu, Kai Zhang, Changhua Zhang, Yulong He

**Affiliations:** 1 Center of Digestive Disease, The Seventh Affiliated Hospital of Sun Yat-sen University, Shenzhen, Guangdong 518107 China; 2 Department of Thoracic Surgery, The Third Affiliated Hospital of Sun Yat-sen University, Guangzhou, China; 3 Center of Gastrointestinal Surgery, The First Affiliated Hospital of Sun Yat-sen University, Guangzhou, China

**Keywords:** MEX3A, Colorectal cancer, EMT, Metastasis, Wnt/β-catenin

## Abstract

**Background:**

Mex-3 RNA binding family members are well-established to be important in cancer development and progression. However, the functions of Mex-3 RNA binding family member A (MEX3A) in colorectal cancer (CRC) metastasis remain poorly understood. In this study, we aim to reveal the function and the mechanism of MEX3A in promoting CRC metastasis.

**Methods:**

We used multiple databases including TCGA database, UALCAN, LinkedOmics, CancerSEA, GeneMANIA and STRING database to investigate the expression, the functions and underlying molecular mechanism of MEX3A in CRC. Multiple experimental methods were adapted to determine the study, including real-time PCR (qPCR), immunohistochemistry (IHC), western blot (WB), transfection, transwell migration and invasion assays, immunofluorescence (IF).

**Results:**

We found that MEX3A was significantly upregulated and correlated to tumor stage and lymph nodal metastasis in CRC through bioinformatics analysis and tissue immunohistochemistry (IHC). The higher expression of MEX3A in CRC correlated with poor recurrence-free survival (RFS) and overall survival (OS). In vitro studies showed that knockdown of MEX3A suppressed EMT transition, invasion and metastasis of CRC cells. Mechanistically, we revealed that MEX3A promotes epithelial-mesenchymal transition (EMT), invasion and metastasis of CRC cells by upregulating the Wnt/β-catenin signaling pathway.

**Conclusion:**

In conclusion, our study reveals that MEX3A promotes CRC migration, invasion and EMT via regulating the Wnt/β-catenin signaling pathway and could be a novel therapeutic target for this patient population.

**Supplementary Information:**

The online version contains supplementary material available at 10.1007/s00432-024-05845-9.

## Introduction

Colorectal cancer is well-established as the third most predominant cancer and the second leading cause of death globally(Bray et al. 2024). In patients with CRC, metastasis is still the leading cause of death. Although the early diagnosis and the survival rates of CRC have gradually improved, metastasis remains the biggest obstacle in CRC treatment(Allemani et al. 2018). It is essential to reveal the mechanisms of CRC metastasis to refine current treatment approaches.

Mex-3 RNA binding family member has 4 homologous isoforms, including MEX3A, MEX3B, MEX3C and MEX3D. All four Mex-3 proteins consist of two K homology (KH) domains and a ubiquitin E3 ligase RING domain(Lederer et al. 2021). This family plays an important role in cancer development and progression, including positive regulation of proliferation, migration, stem cell and immunotherapy resistance(Huang et al. 2018; Jasinski-Bergner et al. 2020). As for MEX3A, an increasing body of evidence suggests it promotes tumorigenesis and development in different cancer. In bladder cancer, the increase expression of MEX3A inhibit cell apoptosis and enhance cell proliferation (Huang et al. 2017). And it promotes breast cancer metastasis and maintaining the stemness via regulating Wnt/β-catenin signaling pathway (Wang et al. 2021b). Recent study reaveled that MEX3A promotes nasopharyngeal carcinoma cell proliferation, invasion, and migration by regulating NF-κB signaling pathway(Xiang et al. 2022). Studies also suggested MEX3A promotes proliferation, migration and invasion in osteosarcoma (Wang et al. 2021a), glioma(Yang et al. 2021), and pancreatic ductal adenocarcinoma(Wang et al. 2020).

As for intestinal tract, previous study revealed that MEX3A is crucial for maintaining the Lgr5 + intestinal stem cells (ISC) pool in the intestinal and critically contributes to intestinal homeostasis(Pereira et al. 2020); mechanistically, the expression of MEX3A in intestinal cell decreased the peroxisome proliferator-activated receptor (PPAR) signaling pathway and induces Wnt signaling pathway(Pereira et al. 2020). Another study revealed that MEX3A regulates caudal type homeobox 2 (CDX2) and impairs intestinal differentiation and cellular polarization(Pereira et al. 2013). These findings suggest that MEX3A plays an essential role in CRC proliferation, invasion or migration(Pereira et al. 2013, 2020). Rencent study showed MEX3A marks drug-tolerant persister cells that mediate relapse after chemotherapy in CRC(Alvarez-Varela et al. 2022). In addition, study revealed MEX3A regulate CRC stem cell self-renewal and differentiation(Yang et al. 2022). And MEX3A promotes migration, invasion, and proliferation through the RAP1/MAPK signaling pathway in CRC(Li et al. 2021). Hitherto, the function and underlying mechanisms of MEX3A in CRC remain poorly understood.

In this study, we committed to reveal the function and the underlying mechanism of MEX3A in CRC. We provided hitherto undocumented evidence that MEX3A promoted EMT, invasion and metastasis of CRC cells by activating the Wnt/β-catenin signaling pathway, suggesting that MEX3A could be a new therapeutic target in CRC.

## Materials and methods

### Bioinformatics analysis

The public databases used included UALCAN database, LinkedOmics database, CancerSEA database, GeneMANIA database, STRING database and TCGA database. The detailed information of these public databases were showed in Supplementary Material.

### Patients

Patients with pathologically confirmed CRC treated with surgery without neoadjuvant at the First Affiliated Hospital of Sun Yat-sen University between 2008 and 2011 were included in this study (*n* = 219). The TNM staging of all cases was restaged according to the 8th edition of the American Joint Committee on Cancer (AJCC8) staging manual. In addition, we randomly selected 80 adjacent normal colon tissues from the 219 patients for paired sample analysis. And we collected 24 samples of paired CRC tissue and normal colon tissue from CRC pateints who accepted surgery treatment in the Seventh Affiliated Hospital of Sun Yat-sen University. This study was approved by the Ethics committee of the Seventh Affiliated Hospital of Sun Yat-sen University.

### Cell culture

The human CRC cells SW480, HCT116 and HT29 were purchased from the Shanghai Institute of Cell Biology, China. The SW480, HT29, and lentivirus-packed cells 293T cells were cultured in DMEM medium supplemented with 10% fetal bovine serum (FBS) (GIBCO, USA). The HCT116 cells were grown in RPMI-1640 media that included 10% fetal bovine serum (FBS) (GIBCO, USA). All cells were cultivated in an incubator under standard conditions (37 °C, 95% humid air and 5% CO_2_).

### Cell transfection, lentivirus production and infection

The full-length human MEX3A cDNA and pCMV-empty vector were purchased from Sino Biological (Beijing, China). The OE-MEX3A plasmids were transfected to cells with lipo6000 and cultivated at 37 °C for 48 h. After 48 h, the transfected cells were collected for RT-PCR, protein expression extract and functional experiments.

The shRNA plasmids specifically targeting MEX3A were purchased from Jikai gene (Shanghai, China). The sequences used are as follows:

- shMEX3A#1, 5′-CGCAAGCCATCCGAATATT-3′;
- shMEX3A#2, 5′-AACCAACACATACATTATC-3′;
- shMEX3A#3, 5′-GCAAGGCTGCAAGATTAAG-3′;
- shCtrl, 5′-TTCTCCGAACGTGTCACGT-3′.

The three plasmids (pspax2, pmd2g and shMEX3A) were co-transfected to 293T cells by lipo6000 (Beyotime, China), according to the manufacturer’s instructions, and shMEX3A lentivirus was collected 48 h after transfection. Subsequently, the lentivirus was added to SW480 and HT29 cells at a density of 3 × 10^5^cells/well, and the transfected cells were divided into two experimental groups, shCtrl and shMEX3A. The cells were cultured with puromycin (2 mg/mL) 48 h after transfection for 1 week.

### Cell migration and invasion assays

Migration and invasion assays were detected using Boyden chambers (Corning). For the migration assay, 1 × 10^5^ HCT116, 2 × 10^5^ SW480 and HT29 cells were seeded in upper chambers with 200 µl serum-free medium, and 600 µl medium supplemented with 20% FBS was added to the lower chambers. For the invasion assays, the upper chambers were coated with Matrigel (BD Biosciences, USA), and 2 × 10^5^ HCT116, 3 × 10^5^ SW480 and HT29 cells were seeded. After 48 h of culture, the cells were fixed with 4% PFA and stained with 1% crystal. The cells were counted and imaged using an inverted microscope.

### Quantitative real-time PCR

Total RNA was extracted from colon cancer cell lines or paired colorectal cancer tissue using TRIzol reagent (ThermoFisher). After evaluation of yield and quality, the RNA was reverse transcribed into cDNA following the manufacturer’s instructions (Accurate biology). qPCR was conducted using SYBR Green Premix Pro Taq HS qPCR Kit (Accurate biology), and quantification of mRNA expression was performed on a BioRAD Real-Time PCR System.

### Western blotting

Total proteins were extracted from cells using lysis buffer with protease inhibitors and PMSF. Nucleoprotein and Cytoplasmic protein were extracted from cells by Nuclear and Cytoplasmic Protein Extraction Kit (Beyotime) according to the manufacturer’s instructions. After quantification using the bicinchoninic acid (BCA) method, the same amounts of total proteins were loaded on each lane and separated by SDS/PAGE. Subsequently, the isolated proteins were transferred into the PVDF membranes. The membranes were blocked with 5% BSA for 1 h and incubated with the corresponding antibody at 4 °C overnight. Primary antibodies consisted of anti-MEX3A polyclonal rabbit antibody (1:1000, Abcam, #ab79046), anti-E-cadherin monoclonal mouse antibody (1:2000, Proteintech, #60,335), anti-N-cadherin monoclonal mouse antibody (1:2000, Proteintech, #66,219), anti-Vimentin monoclonal mouse antibody (1:1000, Proteintech, #60,330), anti-β-catenin monoclonal rabbit antibody (1:1000, CST, #8480S), c-Myc Rabbit Polyclonal Antibody (1:2000, Proteintech, #10,828) and anti-Phospho-β-Catenin (Ser675) monoclonal rabbit antibody (1:1000, CST, # 4176 S). The membranes were incubated with secondary antibodies at room temperature for one hour after being washed with TBST. Finally, bands were imaged using the BioRAD Imaging System with a chemiluminescence detection kit (ECL) assay.

### Immunohistochemical (IHC) staining

Paraffin-embedded CRC specimens used in this study were provided by the Department of Pathology of First Affiliated Hospital of Sun Yat-sen University. Paraffin specimen sections were heated at 80 ℃ for three hours, then dewaxed in xylene and dehydrated with gradient ethanol. 3.0% H_2_O_2_ was used to block peroxide for 15 min, and antigens were repaired in citric acid buffer with water bath heating for 40 min. After goat serum blocking, the slides were incubated with anti-MEX3A (1:400, Abcam, ab79046) at 4 °C overnight. The secondary, HRP-linked secondary antibodies incubated for 40 min and DAB were used to visualize the bound primary antibodies. Semiquantitative scoring method was adopted(Li et al. 2019). No staining or less than 10% of the tumor cells stained was scored as IHC 0. When the percentage of tumor cells stained exceeded 10%, IHC was scored using staining intensity and as follows: 1 (weak), 2 (moderate) and 3 (strong). Scores of 0 and 1 were defined as low, and scores of 2 and 3 were defined as high MEX3A expression.

### Immunofluorescence assay

3 × 10^4^ cells were seeded in a chamber with glass coverslips for 24 h, then fixed with 4% paraformaldehyde for 20 min. After three times of PBS washing, 0.1% Triton X-100 was added to permeate the cells. After additional PBS washing, the cells were blocked with goat serum for 30 min at room temperature and incubated with corresponding antibodies overnight at 4°C. The following day, the cells were incubated with the corresponding fluorescent secondary antibody for 1 h at room temperature. After PBS washing, DAPI was used for staining the nucleus. All images were generated by a Leica Microscope.

### Statistical analyses

The Chi-square and Wilcoxon rank-sum tests were used for categorical and continuous variables, and the independent sample T-test was used for continuity variables. A p-value < 0.05 was statistically significant. Statistical analysis software included SPSS statistical software (version22.0; SPSS Inc., Chicago, IL) and GraphPad Prism (version 8.0). *P < 0.05, ** *P* < 0.01, ***P < 0.001.

## Results

### MEX3A expression is upregulated in CRC tissues

**Fig. 1:**
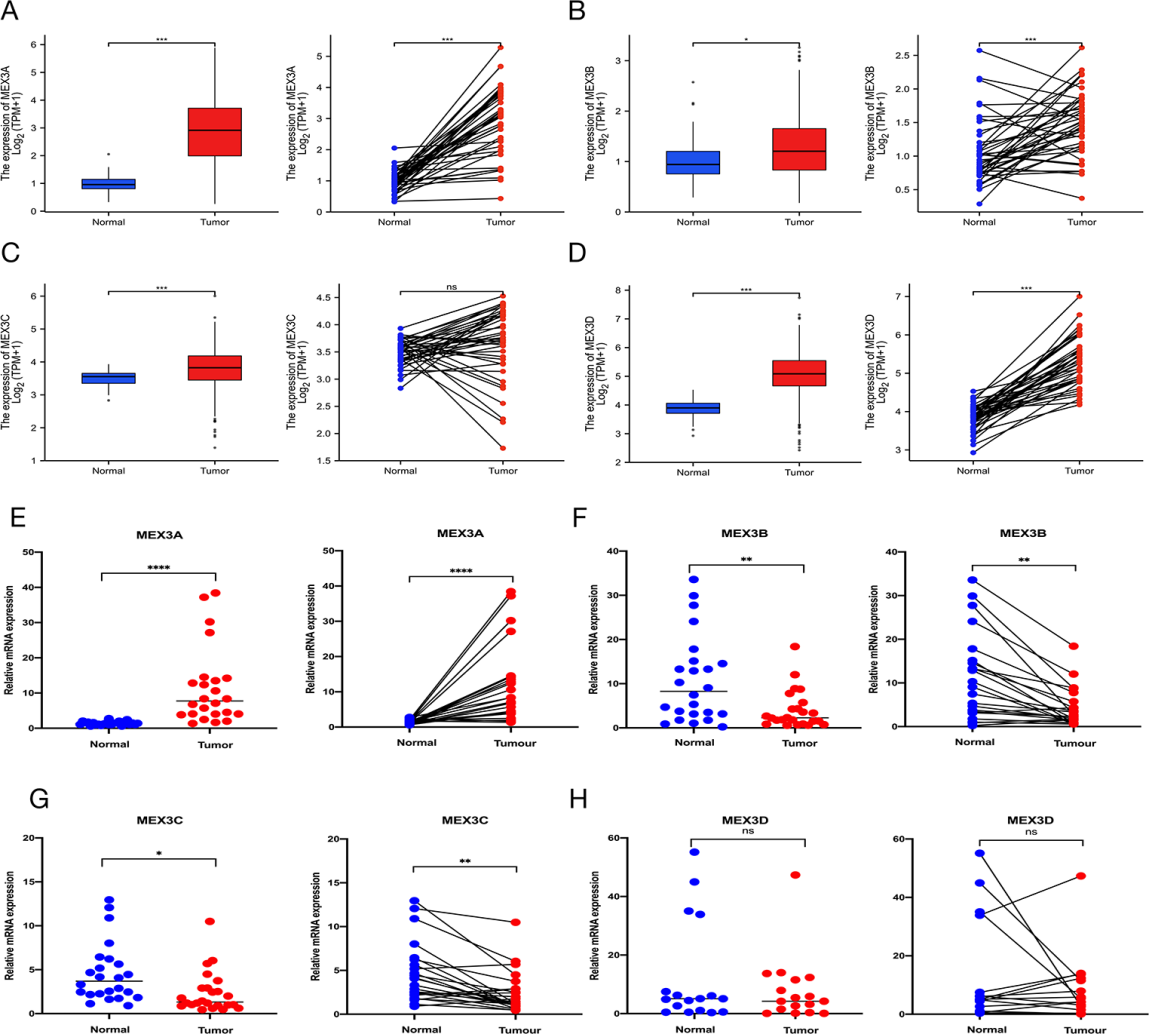
MEX3A expression is upregulated in CRC tissues. (**A**-**D**) The mRNA expression of MEX3A, MEX3B, MEX3C and MEX3D in CRC and its paired normal tissue. (**E**-**H**) RT-PCR results of 24 pairs mRNA expression of MEX3 family in CRC patient. The mRNA expression of MEX3A, MEX3B, MEX3C and MEX3D in CRC and its paired normal tissue

To explore the role of the MEX3 family in cancers, we used TCGA for pan-cancer analysis of the expression of MEX3A, MEX3B, MEX3C and MEX3D. The results showed that all 4 members of the MEX3 family exhibited different degrees of upregulated expression in various cancers, including CRC (Figures S1A-D). This result indicated that the MEX3 family might play a pro-carcinogenic role in many cancers. Then we further analyzed the expression of these 4 members in CRC. The result showed that MEX3A, MEX3B and MEX3D were highly expressed in CRC compared with normal tissue in paired and unpaired analyses, while the expression of MEX3C was not statistically significant in paired analysis (Fig. 1A-D). To validate the results, we extracted RNA from 24 samples of paired CRC tissue and normal colon tissue from surgical specimens and performed RT-PCR. Consistent with TCGA data, MEX3A was highly expressed in tumors compared with normal tissue in the paired and unpaired analysis. Moreover, MEX3A was significantly upregulated (Fig. 1E), the expression of MEX3B and MEX3C were decreased (Fig. 1F-G), and the expression of MEX3D was comparable to adjacent normal tissue (Fig. 1H). These results suggested that MEX3A could play a pro-carcinogenic role in CRC.

### High expression of MEX3A correlated with poor prognosis

**Fig. 2:**
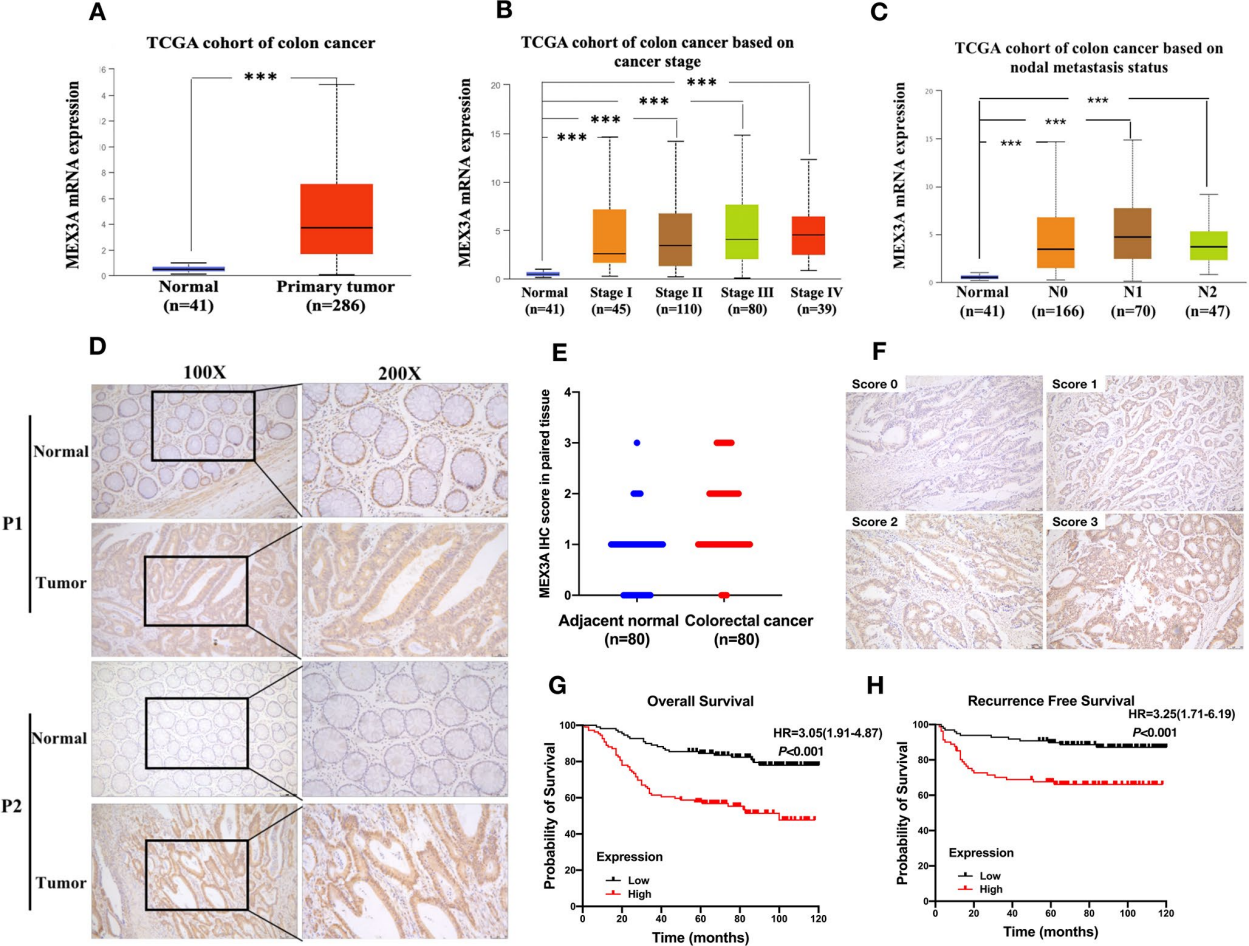
Upregulated MEX3A expression is associated with poor prognosis in CRC. (**A**) UALCAN datebase showed MEX3A mRNA expression were significant inceresaed in colon cancer (*P* < 0.05). (**B**-**C**) The high expression of MEX3A is associated with tumor stage and lymph node metastasis in colon cancer(*P* < 0.05). (**D**) The MEX3A protein expression in CRC and paired normal colorectal tissue. (**E**) Protein expression of MEX3A was significantly increase in CRC tissue compared with adjacent normal tissues.(**F**)Immunohistochemical score criteria according to the staining degree. Score 0: blue staining, Score 1: light yellow staining, Score 2: brown staining and Score3: dark brown staining. (**G**) The relationship between MEX3A expression in CRC and OS. Log-rank *P* < 0.001. (**H**) The relationship between MEX3A expression in CRC and RFS. Log-rank *P* < 0.001

We used the UALCAN database to explore the expression of MEX3A and its associated clinicopathological parameters. We found that MEX3A high expression in colon cancer tissue than normal tissue (Fig. 2A). The results showed that high expression of MEX3A was positively associated with tumor grade, stage and lymph node metastasis (Fig. 2B and C). Next, we stained 219 CRC specimen sections by IHC and performed univariate analysis, Cox proportional-hazards regression analysis and Kaplan-Meier analysis. The patients were divided into MEX3A high expression group and MEX3A low expression group according to the IHC scores. The results showed that MEX3A was primarily expressed in the cell nucleus and cytoplasm. We examined MEX3A expression in 80 paired CRC tissue samples and the corresponding adjacent tissues. The result showed the protein expression of MEX3A was significantly higher in CRC tissue than normal tissue (Fig. 2D and E, *P* < 0.05). Univariate analysis showed that high expression of MEX3A was associated with gender, tumor differentiation, depth of invasion, lymph node metastasis, distant metastasis and vessel or nerve invasion (Table 1, *P* < 0.05). Moreover, KM analysis indicated that high expression of MEX3A correlated with a poor prognosis in patients with CRC (OS, HR:3.05 95% CI 1.91–4.87, *P* < 0.001; RFS, HR:3.23 95% CI 1.71–6.19, *P* < 0.001, respectively) (Fig. 2G and H). Futher Cox proportional-hazards regression analysis showed that MEX3A was an independent risk factors for OS (HR: 2.132, 95% CI 1.254–3.625, *P* = 0.002) and for RFS (HR:3.021 95% CI 1.142–5.904 *P* = 0.002) (Tables 2 and 3). Taken together, the data indicated that MEX3A high expression predicted a worse survival and was an independent prognostic factor for the patients with CRC.

**Table 1:** Associations of MEX3A expression with clinical parameters in 219 CRC patients

Characteristic	No.	MEX3A expression		*P* value
		Low (*N* = 110)	High (*N* = 109)	
**Age (year)**		59.9 ± 14.0	58.7 ± 13.1	0.091
≤ 60y	102	45	57	
> 60y	117	65	52	
**Gender**				0.018
Male	122	70	52	
Female	97	40	57	
**Tumor location**				0.553
Right hemicolon	115	56	59	
Left hemicolon	104	54	50	
**Differentiation**				0.001
Well + Moderate	151	88	63	
Poor	68	22	46	
**Depth of invasion**				0.048
T1	2	2	0	
T2	24	15	9	
T3	136	72	64	
T4	57	21	36	
**Lymph node metastasis**				0.003
N0	147	85	62	
N1	52	20	32	
N2	20	5	15	
**Distant metastasis**				0.005
M0	179	98	81	
M1	40	12	28	
**TNM stage**				0.000
I	21	16	5	
II	109	65	44	
III	49	17	32	
IV	40	12	28	
**CEA level (µg/L)**				0.952
≤ 5	123	62	61	
> 5	96	48	48	
**CA19-9 level (U/ml)**				0.133
≤ 35	155	83	72	
> 35	38	17	25	
**Vessel or nerve invasion**				0.001
Yes	41	11	30	
No	177	110	109	
**Chemotherapy or not**				0.156
Yes	115	63	52	
No	104	47	57	

**Table 2:** Cox proportional-hazard regression analysis for Overall Survival in 219 patients with CRC

	Univariate analysis				Multivariate analysis			
	*P*-value	HR	95.0% CI for Exp (B)		*P*-value	HR	95.0% CI for Exp (B)	
			Lower	Upper			Lower	Upper
**Gender**	0.941	0.983	0.619	1.560				
**Age**	0.930	1.021	0.664	1.617				
**Tumor location**	0.324	1.010	0.636	1.605				
**Differentiation**	0.000	5.109	3.184	8.192	0.197	1.616	0.780	3.349
**Depth of invasion**	0.015	5.857	1.628	21.075	0.061	6.713	0.915	24.625
T1 + T2								
T3 + T4								
**Lymph node metastasis**	0.000	2.580	1.629	4.087	0.024	1.805	1.081	3.014
N0								
N+								
**Distant metastasis**	0.000	6.750	4.173	10.920	0.006	2.712	1.336	5.503
**TNM stage**	0.000	3.993	2.458	6.448				
I + II								
III + IV								
**CEA level**	0.045	1.602	1.011	2.537	0.888	1.038	0.619	1.742
**CA19-9 level**	0.101	1.554	0.917	2.632				
**Vessel or nerve invasion**	0.000	5.940	3.694	9.551	0.028	1.989	1.077	3.670
**Chemotherapy or not**	0.521	0.859	1.541	1.366	0.001	2.357	1.441	3.855
**MEX3A expression**	0.000	3.122	1.888	5.164	0.002	2.132	1.254	3.625
Low								
High								

**Table 3:** Cox proportional-hazard regression analysis for Recurrence Free Survival in 219 patients with CRC

	Univariate analysis				Multivariate analysis			
	*P*-value	HR	95.0% CI for Exp (B)		*P*-value	HR	95.0% CI for Exp (B)	
			Lower	Upper			Lower	Upper
**Gender**	0.314	1.382	0.736	2.592				
**Age**	0.726	0.893	0.476	1.677				
**Tumor location**	0.295	0.708	0.372	1.351				
**Differentiation**	0.009	2.540	1.263	5.108	0.216	1.672	0.740	3.776
**Depth of invasion**	0.051	7.248	0.995	52.795				
T1 + T2								
T3 + T4								
**Lymph node metastasis**	0.020	2.140	1.130					
N0								
N+								
**TNM stage**	0.017	2.240	1.133	4.064	0.736	1.147	0.516	2.550
I + II								
III + IV								
**CEA level**	0.043	1.913	1.019	3.591	0.049	1.912	1.002	3.647
**CA19-9 level**	0.626	1.228	0.538	2.804				
**Vessel or nerve invasion**	0.000	4.279	1.957	9.353	0.023	2.597	1.142	5.904
**Chemotherapy or not**	0.236	1.463	0.780	2.747	0.941	1.026	0.513	2.055
**MEX3A expression**	0.001	3.252	1.644	6.431	0.002	3.021	1.142	5.904
Low								
High								

### MEX3A promotes CRC cell migration and invasion in vitro

**Fig. 3:**
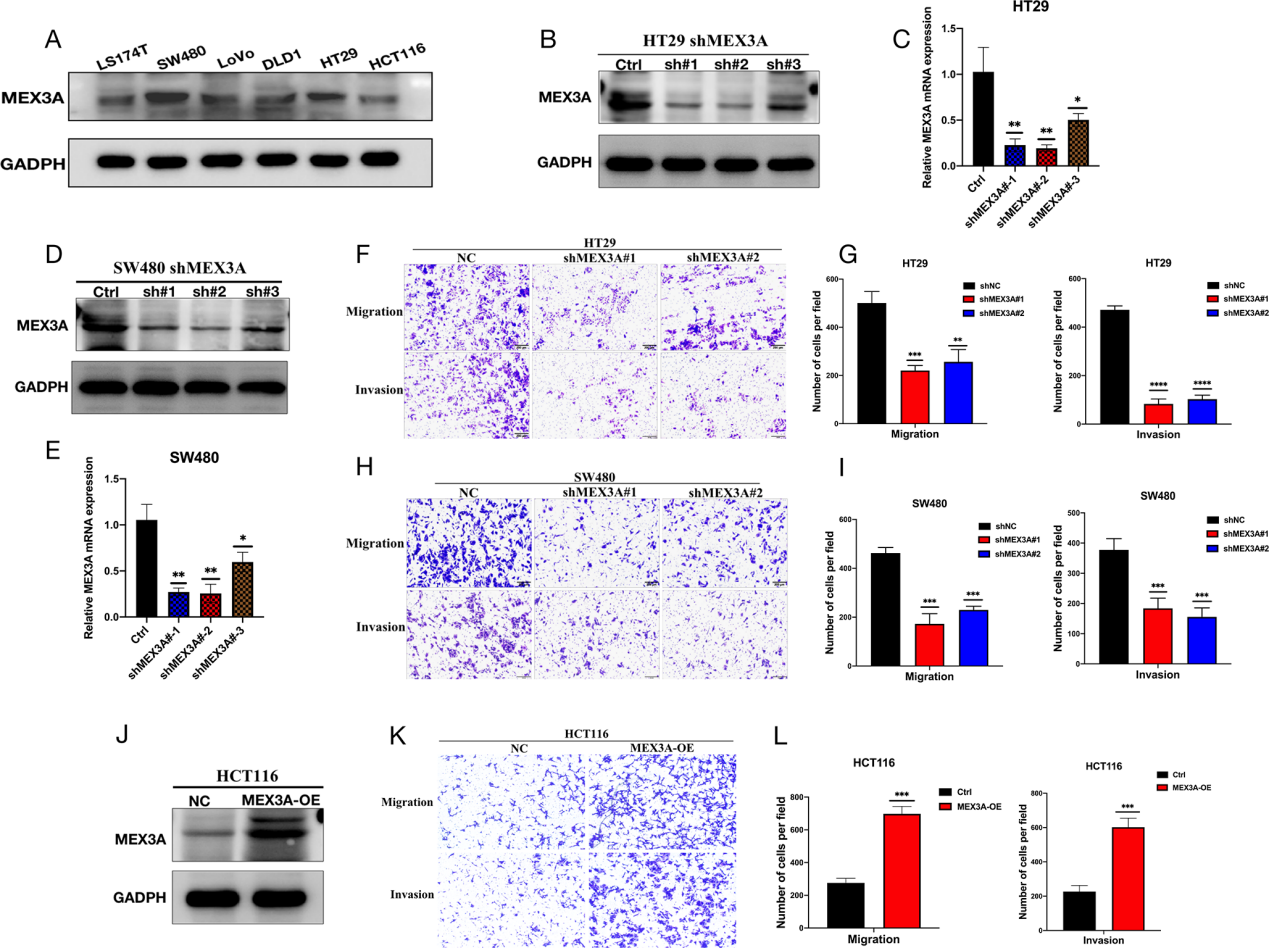
MEX3A promotes CRC cell migration and invasion in vitro. (**A**)The expression of MEX3A in CRC cell lines. (**B**-**C**)The mRNA and protein levels after effective MEX3A knockdown in HT29 cells. (**D**-**E**) The mRNA and protein levels after effective MEX3A knockdown in SW480 cells. (**F**-**G**) Migration and invasion ability of HT29 cells measured by Transwell experiment. (**H**-**I**) Migration and invasion ability of SW480 cells measured by Transwell experiment. (**J**) The protein level in MEX3A overexpressing HCT116 cells. (**K**-**L**) Migration and invasion ability of HCT116 cells measured by Transwell experiment

Our previous study results indicated that MEX3A is a pro-carcinogenic gene in CRC. To reveal which functions of CRC cells are mediated by MEX3A, MEX3A was knocked down and overexpressed in CRC cells. The WB of CRC cell lines showed relatively high expression of MEX3A in HT29 and SW480 cells and relatively low expression in HCT116 cells. The WB results for MEX3A protein levels were consistent with the PCR results (Fig. 3A). Next, we chose HT29 and SW480 cell lines to perform MEX3A knockdown by lentivirus and overexpressed MEX3A in the HCT116 cell line by plasmid transfection. The effect of interference expression was verified by qPCR and WB. Both qPCR and WB exhibited effective knowndown after transfection of shMEX3A#1 and shMEX3A#2 in HT29 and SW480 cells (Fig. 3B-E). The migration and invasion abilities were significantly weakened after the knockdown of MEX3A in HT29 and SW480 cells assessed by the transwell assay (Fig. 3F-I). Consistently, when MEX3A was overexpressed in HCT116 cells, the cell migration and invasion abilities were enhanced (Fig. 3J-L). These results indicated that MEX3A promotes CRC progression by enhancing cancer cell migration and invasion.

### The potential function and signal pathway of MEX3A in CRC

**Fig. 4:**
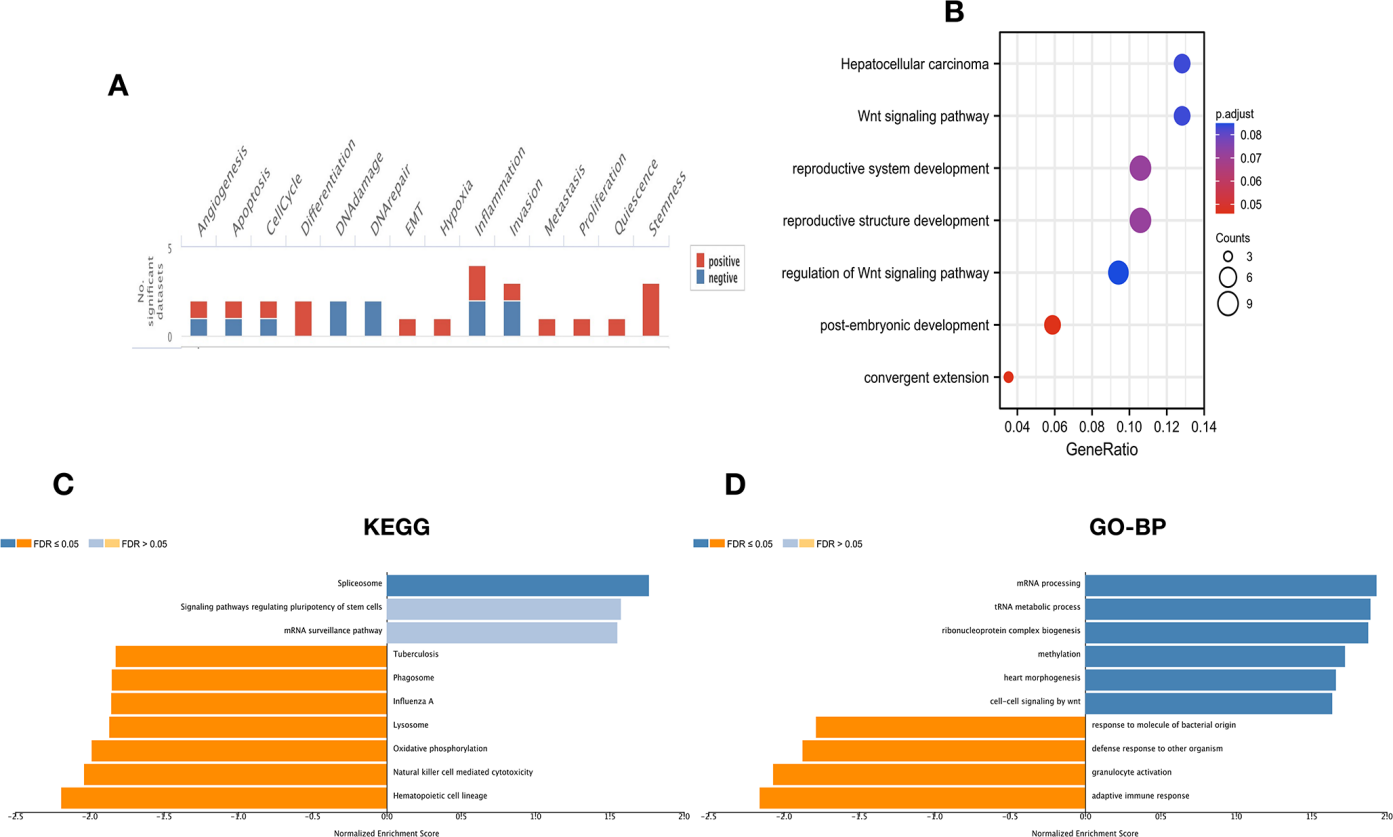
The potential function and signal pathway of MEX3A in CRC. (**A**) The function of MEX3A measured by CancerSEA database. (**B**) KEGG enrichment of MEX3A function and involved signal pathway in CRC. (**C**, **D**) KEGG enrichment and biological process of MEX3A in CRC measured by LinkedOmics database

To reveal the mechanism of MEX3A in CRC cell migration and invasion, we used LinkedOmics and CancerSEA database to explore the potential function and signal pathway of MEX3A. CancerSEA database analysis showed that MEX3A was involved in stemness, metastasis, invasion and EMT (Fig. 4A). Consistently, LinkedOmics datebase showed MEX3A was associated to stem cells and the Wnt signal pathway (Fig. 4C-D). Next, we used TCGA database conducted KEGG pathway analysis and showed that MEX3A regulated the Wnt signal pathway. Taken together, the above results suggest that MEX3A promotes CRC cell migration and invasion by regulating the Wnt signal pathway (Fig. 4B). Another interesting finding is that KEGG and GO analyses (biological process) of MEX3A showed natural killer cell-mediated cytotoxicity and adaptive immune response (Fig. 4C and D). Further investigations showed that MEX3A in CRC was negatively correlated to immune cell infiltration, including CD4 + T cell, CD8 + T cell, DC, Macrophage, MDSC, NK cell, Th17 cell, Treg and B cell. Moreover, the expression of MEX3A was negatively correlated to immune checkpoint gene expression in CRC, including PDCD1, PDCD1LG2, CTLA4, LAG3, HAVCR2, and CD274 (Figure S2).

### The potential regulatory network of MEX3A in CRC

**Fig. 5:**
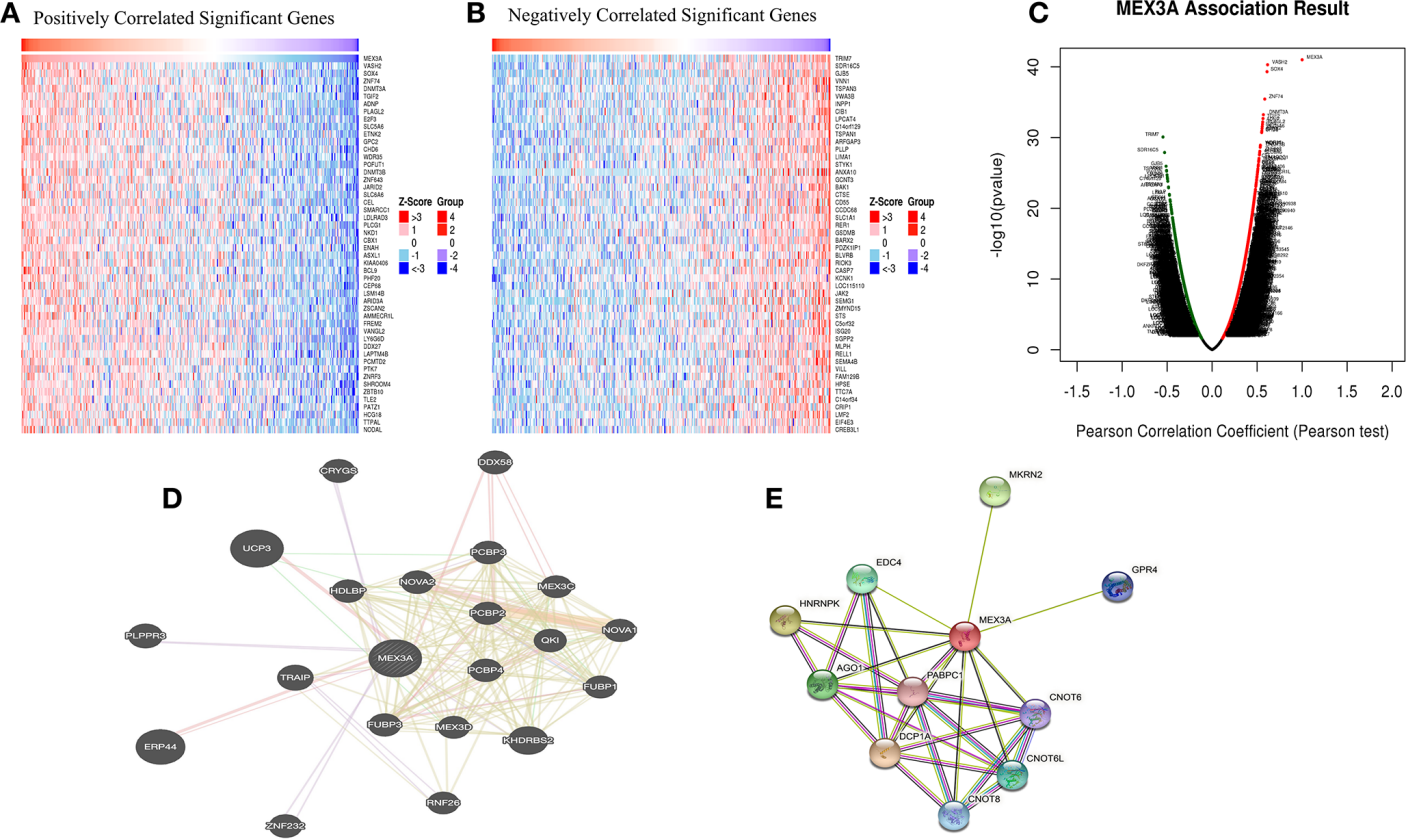
The potential regulation network of MEX3A in CRC. (**A**, **B**) The top 50 positively and negatively regulated genes associated with MEX3A in CRC are shown in heatmaps. Red means positive, and blue means negative. (**C**) Volcano plot showing the positive and negative genes relative to MEX3A in CRC. (**D**, **E**) The gene-gene and protein-protein networks of MEX3A plotted by GeneMANIA database and STRING database

To further understand the potential regulatory role of MEX3A in promoting CRC cell invasion and metastasis, we used LinkedOmics to investigate the positive and negative co-expression genes associated with MEX3A in CRC. The top 50 positive and negative co-expression genes are shown in the heatmap and volcano plot (Fig. 5A-C). The most positive correlation gene associated with MEX3A in CRC were VASH2 and SOX4. Furthermore, TRIM7 exhibited the strongest negative correlation. In addition, the gene-gene and protein-protein interaction networks of MEX3A were plotted by the GeneMANIA database and STRING database, respectively (Fig. 5D-E).

### MEX3A regulates CRC cell EMT transition and wnt signal pathway

**Fig. 6:**
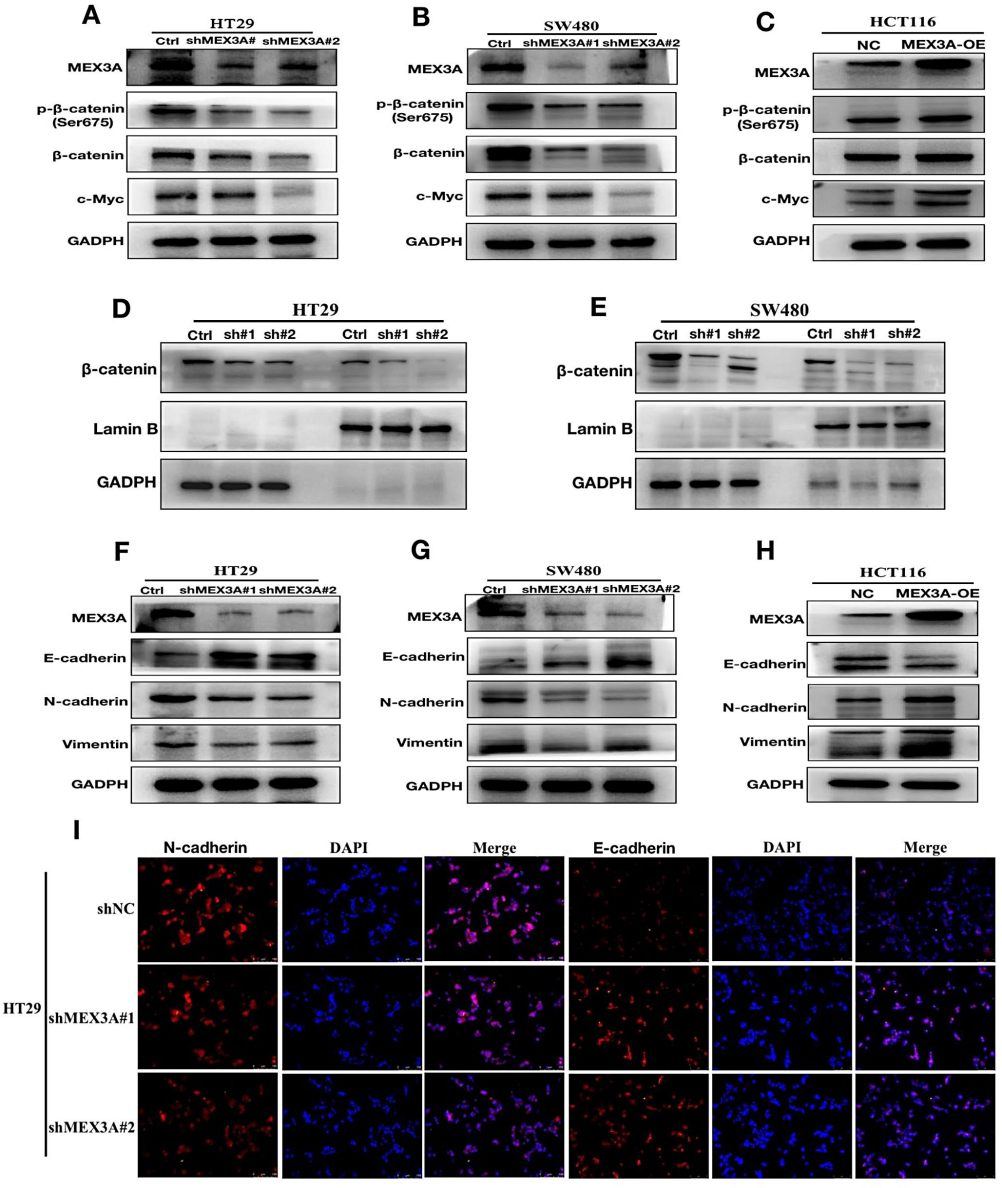
MEX3A regulates CRC cell EMT transition and Wnt signal pathway. (**A**, **B**) β-catenin, c-Myc and phosphorylation β-catenin were decreased after the knockdown of MEX3A in HT29 and SW480 cell lines. (**C**) The β-catenin, phosphorylation β-catenin and c-Myc were increased when MEX3A was overexpressed in HCT116. (**D**, **E**) The β-catenin in the nucleus was decreased after the knockdown of MEX3A. (**F**, **G**)N-cadherin and Vimentin were decreased, and E-cadherin was increased after the knockdown of MEX3A in HT29 and SW480 cells. (**H**) N-cadherin and Vimentin were increased, and E-cadherin decreased in MEX3A-overexpressing HCT116 cells. (**I**) Immunofluorescence showed N-cadherin and E-cadherin changes with knockdown of MEX3A in HT29 cells

To validate our hypothesis that MEX3A promotes CRC cell migration and invasion by regulating the Wnt signal pathway, we investigated changes in the signal pathway and protein in MEX3A knockdown cells and MEX3A overexpression cells. The results showed that β-catenin, c-Myc and phosphorylation β-catenin were decreased when MEX3A was knocked down in HT29 and SW480 cell lines (Fig. 6A and B). In addition, the β-catenin and c-Myc were increased when MEX3A was overexpressed in HCT116 (Fig. 6C). Consistently, the β-catenin in the nucleus also decreased (Fig. 6D and E). A previous study revealed that MEX3A regulates CDX2 and impairs intestinal differentiation and cellular polarization by regulating E-cadherin (Pereira et al. 2013). We further investigated whether MEX3A regulates EMT transition in CRC cells, which plays an important role in tumor metastasis. The results showed decreased N-cadherin and Vimentin levels and increased E-cadherin after MEX3A was knocked down in HT29 and SW480 cells (Fig. 6F, G and H). Consistently, the overexpression of MEX3A in HCT116 cells caused an increase in N-cadherin and Vimentin and decreased E-cadherin (Fig. 6F). Taken together, our results suggest that MEX3A promotes CRC cell migration, invasion and EMT transition by regulating the Wnt/β-catenin signal pathway.

## Discussion

Metastasis remains the main cause of mortality in CRC and the biggest obstacle to curative treatment. Cancer metastasis is reportedly responsible for ~ 90% of cancer-associated mortality(Lambert et al. 2017; Ganesh and Massagué 2021). Further studies are warranted to uncover the mechanism of metastasis and develop targeted therapy accordingly.

In this study, we comprehensively studied the expression of the MEX-3 RNA binding family members in CRC and showed that only MEX3A was upregulated and correlated with a poor prognosis. Mechanistically, overexpression of MEX3A enhanced CRC metastasis by promoting EMT transition and the Wnt/β-catenin signaling pathway.

Previous studies have revealed that MEX-3 RNA binding family members play an important role in cancer development and progression, especially MEX3A. A recent study revealed that MEX3A marks a subpopulation of Lgr5 + intestinal stem cells and MEX3A-expressing cells resistant to chemotherapy and γ-radiation in the intestinal epithelium (Barriga et al. 2017). Further studies indicated that MEX3A regulates Lgr5 + stem cells, which maintain the development of the intestinal epithelium by regulating the PPAR pathway and knockdown the expression of MEX3A suppresses the Wnt signaling pathway(Pereira et al. 2020). These findings indicated that MEX3A might play an important role in CRC development. However, the role of MEX3A in CRC development remains unclear. This study provided compelling evidence that MEX3A promotes CRC cell metastasis and EMT via Wnt/β-catenin signaling.

We found that N-cadherin and Vimentin were decreased when MEX3A was knocked down in HT29 and SW480 cells, while E-cadherin levels were increased. The opposite results were observed after overexpression of MEX3A in HCT116 cells. Consistently, a previous study revealed that MEX3A regulates CDX2 and impairs intestinal differentiation and cellular polarization by regulating E-cadherin (Pereira et al. 2013). These evidence supports the conclusion that MEX3A plays a regulatory role in the EMT status of CRC cells. Previous studies showed that EMT drive CRC cell invasion and metastasis(Yan et al. 2015; Amilca-Seba et al. 2022; Kim et al. 2022). An increasing body of evidence from recent studies suggests EMT promote cancer cell invasion and metastasis in various cancers, and inhibite the tumors’ EMT may be a therapeutic strategy.(Krebs et al. 2017; Stemmler et al. 2019). In addition, EMT in cancer can also give rise to a cancer stem cell (CSC) state, which can promote tumor metastasis and drug resistance (Roy et al. 2021; Zheng et al. 2021).

The mechanism of EMT are diverse. Studies showed the Wnt pathway regulates EMT and CSCs, leading to malignant progression, and it has been considered a key activator of EMT. In addition, current evidence suggests that the Wnt/β-catenin signaling pathway is frequently activated in CRC and promotes cancer progression. (Gonzalez and Medici 2014; de Sousa e Melo et al. 2017; Tammela et al. 2017; Nieszporek et al. 2019). This study substantiated that MEX3A promotes CRC invasion and metastasis by regulating EMT via the Wnt pathway. Interestingly, it has been shown that MEX3A regulates the Wnt pathway by downregulating Dickkopf WNT signaling pathway inhibitor 1 (DKK1) expression in breast cancer(Wang et al. 2021b). Furthermore, MEX3A can downregulate CDX2 expression via a post-transcriptional mechanism in the intestinal epithelium (Pereira et al. 2013), and a previous study revealed that CDX2 could suppress Wnt/β-catenin signaling (Yu et al. 2019). Thus, we hypothesized that MEX3A could activate the Wnt pathway by downregulating CDX2. Nonetheless, further research is indicated to provide more evidence on the regulatory mechanism of MEX3A on the Wnt pathway.

Our study used multiple approaches to evaluate the function and mechanism of MEX3A in CRC and the results demonstrated that MEX3A promotes CRC invasion, metastasis and EMT via the Wnt/β-catenin signaling pathway. However, the limitations in our current study is that we haven’t clarified the detailed mechanisms how MEX3A modulates Wnt/β-catenin signaling pathway activation in CRC. Moreover, in vivo study such as mouse models of MEX3A functions is lack. Further research is needed to provide more evidence in the future.

## Conclusions

In conclusion, MEX3A is overexpressed in CRC tissue and associated with a poor prognosis. Mechanistically, MEX3A promotes CRC invasion, migration and EMT transition via the Wnt/β-catenin signaling pathway and might act as a therapeutic target.

### Electronic supplementary material

Below is the link to the electronic supplementary material.

Supplementary Material 1

Supplementary Material 2

## Data Availability

The data showed in this article can be found in online repositories. The detail of repositories can be seen in Materials and Methods of this article and Supplementary Material.
