# Supplementary figures and images for: MEX3A promotes colorectal cancer migration, invasion and EMT via regulating the Wnt/β-catenin signaling pathway
Source: J Cancer Res Clin Oncol. 2024 Jun 25;150(6):319. doi: 10.1007/s00432-024-05845-9 (PMC11196291; doi:10.1007/s00432-024-05845-9)

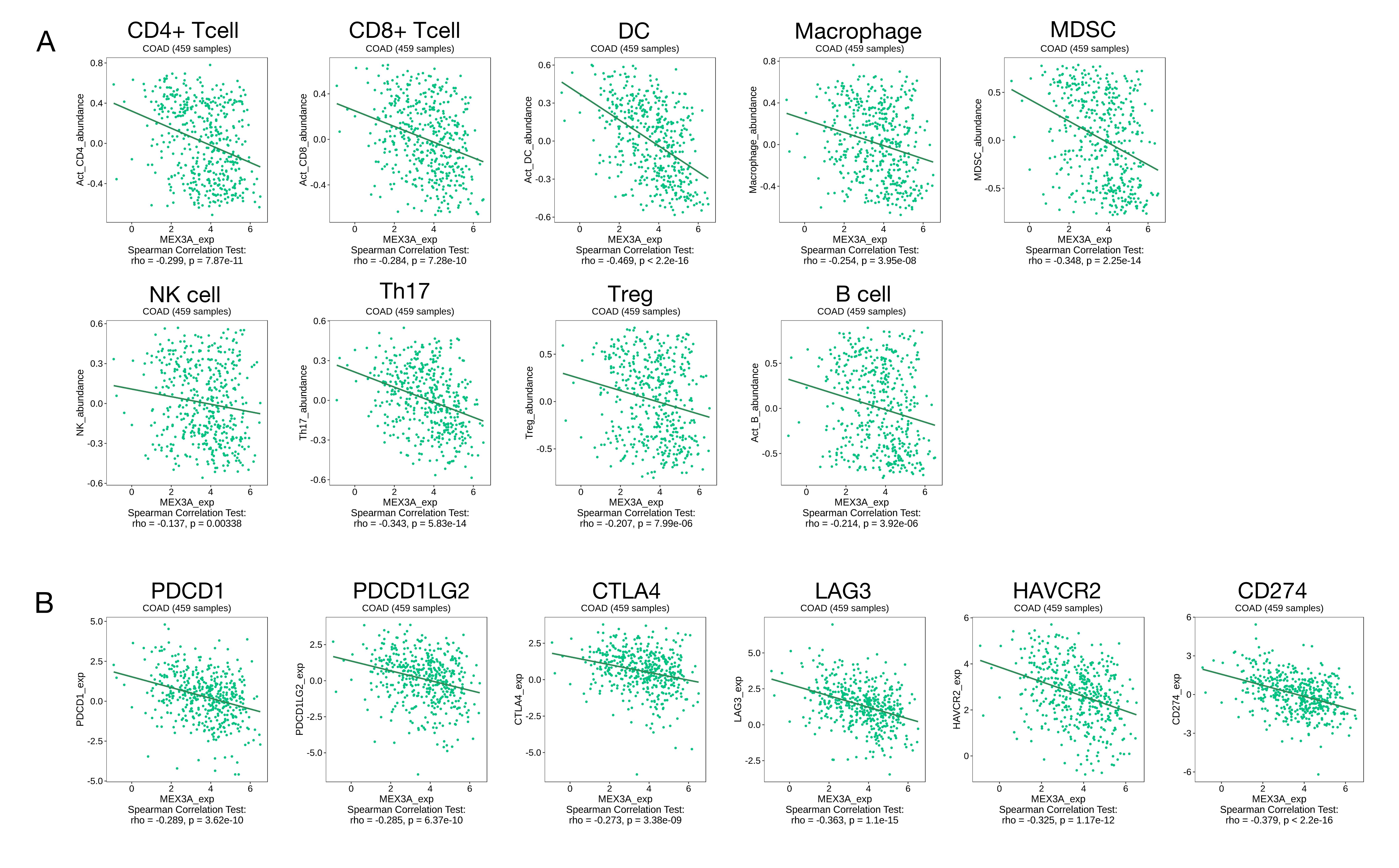

Supplement: Supplementary file 1 — Supplementary Material 1 [file 432_2024_5845_MOESM1_ESM.tif]
